# A real-word study: is normothermic intraoperative intraperitoneal chemotherapy impactful as we expect?

**DOI:** 10.3389/fonc.2023.1172782

**Published:** 2023-07-04

**Authors:** Mengyao Jin, Wei Cao, Junjie Chen, Maoming Xiong, Guodong Cao, Bo Chen

**Affiliations:** ^1^Department of General Surgery, First Affiliated Hospital of Anhui Medical University, Hefei, China; ^2^Department of Surgery, The People’s Hospital of Hanshan County, Ma’anshan, China

**Keywords:** subgroup, lobaplatin IPC = intraperitoneal chemotherapy, NIIC = normothermic intraoperative intraperitoneal chemotherapy, gastric cancer, real-world, regulatory science

## Abstract

**Background:**

Patients with gastric cancer have a poor prognosis. Currently, intraperitoneal chemotherapy has been considered a therapeutic option to improve prognosis due to its appealing theoretical rationales. But there is no consensus on the choice of chemotherapeutic agents used in intraperitoneal chemotherapy for gastric cancer. The real-world efficacy of applying intraoperative chemotherapy in gastric cancer still remains undefined.

**Methods:**

Patients with gastric cancer who underwent radical gastrectomy at the Gastrointestinal Department of The First Affiliated Hospital of Anhui Medical University between 2012 and 2019 were enrolled in this study. Patients were divided into two groups based on whether they received intraperitoneal chemotherapy. The t-test (mean of two samples) was conducted to compare the difference in measurement data between the two groups, and the chi-square test was used to compare the difference in count data. Kaplan-Meier method with log-rank test was performed to analyze the overall survival of patients. Kaplan-Meier method with log-rank test was also performed in various subgroups to respectively compare the survival of patients. Multivariate Cox analysis was performed to analyze the prognosis factors of these patients.

**Results:**

A total of 1253 patients were included in the final analysis, in which 861 patients received intraperitoneal chemotherapy and 352 not received intraperitoneal chemotherapy. The clinicopathological features of the participants in the two groups were comparable. There was no significant difference between the two groups in overall survival (*P* > 0.05). Consistently, no significant difference was found between the two groups in each subgroup (*P* > 0.05). The multivariate Cox analysis demonstrated that only age, BMI, pathological type, TNM stage, and differentiation grade were independent risk factors of survival.

**Conclusion:**

Intraoperative intraperitoneal chemotherapy usage did not improve survival in patients with gastric cancer undergoing radical gastrectomy.

## Introduction

Gastric cancer is among the most common and deadly cancers in the world (1). According to GLOBOCAN 2018 data, gastric cancer remains the third most common cause of cancer-related death and the fifth most diagnosed malignancy globally (2). More than 1 million new cases of gastric cancer are reported annually worldwide (3). Furthermore, the majority of patients miss the surgical opportunity in that they are already in the advanced stage when diagnosed owing to the insidious onset and rapid progression. Despite non-surgical treatments including radical surgery and intravenous chemotherapy have been widely used for gastric cancer, the long-term survival rate still remains unsatisfactory (4). In addition to the harm to physical and mental health, gastric cancer also represents a significant burden on society.

Intraperitoneal chemotherapy (IPC), which is considered to be beneficial for concentrating chemotherapeutic efficacy, has been increasingly used due to the appealing theoretical rationales. Innovative results have shown the necessity to keep increasing consideration into the intraperitoneal administration of chemotherapies. Despite the widespread clinical use of IPC for gastric cancer, its efficacy and safety still remain controversial. Currently, agents applied in IPC include cisplatin, 5-FU, hydroxycamptothecin, Sinofuan, and so on. Intraoperative use of extended-release 5-FU implants is a relatively new approach and has been widely used in almost all types of digestive tract cancers in China (5). But for a section of drugs applied in IPC, there are no guidelines that recommend them as intraperitoneal perfusion drugs for gastric cancer yet. In addition, the drug classification, temperature, concentration, and location may vary among different IPC strategies, while these are closely related to the efficacy of IPC (6). Based on randomized controlled trials (RCT) reporting efficacy of IPC, IPC can be mainly summarized as the following five types: normothermic intraoperative intraperitoneal chemotherapy (NIIC), normothermic postoperative intraperitoneal chemotherapy (NPIC), hyperthermic intraoperative intraperitoneal chemotherapy (HIIC), hyperthermic postoperative intraperitoneal chemotherapy (HPIC) and hyperthermic intraoperative intraperitoneal chemotherapy combined with postoperative intraperitoneal chemotherapy (HIIC+PIC). Nonetheless, there are no international guidelines or expert consensus that can guide peritoneal perfusion therapy. These factors put us into doubt whether IPC truly benefits the prognosis of patients with gastric cancer as we expected.

Over the past few years, an increasing number of studies have drawn optimistic conclusions that IPC can be effective in improving prognosis. Studies are showing that application of NIIC can effectively prevent tumor recurrence and peritoneal metastasis, bringing promising clinical effects. A multi-center, randomized, open-label, controlled clinical study demonstrated that for stage III gastric cancer, intraoperative sustained-release fluorouracil implants after radical resection combined with postoperative adjuvant chemotherapy could significantly reduce the risk of peritoneal recurrence and prolong PFS (7). A meta-analysis by Jin-yu Huang claimed that HIIC and NIIC should be recommended to treat patients with gastric cancer because of improvement in overall survival. However, it was also found that NIIC can increase the risks of marrow depression, intra-abdominal abscesses, and fever (8). Meanwhile, it has also been reported that intraperitoneal administration of mitomycin C or cisplatin resulted in no significant clinical effects against peritoneal metastasis of gastric cancer (9). Apparently, there are different opinions about the exact efficacy of IPC in gastric cancer patients. These studies have the following limitations: insufficient follow-up time, limited sample size, and high rate of loss to follow-up. To explore the real impact of IPC on the prognosis of patients with gastric cancer, we collected single-center large-sample data to complete the real-world study, which mainly focused on the efficacy of NIIC.

## Materials and methods

This study was conducted following the World Medical Association Declaration of Helsinki. This protocol was approved by the institutional review board of The First Affiliated Hospital of Anhui Medical University.

We collected the patient’s clinical characteristics, including gender, age, body mass index (BMI), chronic disease (hypertension or diabetes), lifestyle habits (smoking and drinking), and the treatment of NIIC. According to the revised Asia-Pacific BMI criteria by the World Health Organization (World Health Organization, 2004), BMI was divided into three types, including underweight (BMI<18.5), normal weight (18.5 ≤ BMI ≤ 23.9), and overweight/obesity (BMI ≥ 24.0 kg/m^2^).

Postoperative factors, including the pathological type, differentiation grade, TNM stage (tumors were staged according to the seventh edition of the American Joint Committee on Cancer tumor-node-metastasis staging system), and operative time were also evaluated.

### Participants

A total of 1682 patients diagnosed with gastric cancer underwent surgery at the Gastrointestinal Department of The First Affiliated Hospital of Anhui Medical University between 2012 and 2019 were retrospectively analyzed. Inclusion criteria: (1) a diagnosis of gastric cancer confirmed by preoperative biopsy and postoperative pathology report; (2) no serious heart, lung, liver and kidney dysfunction; (3) a primary treatment without any other chemotherapy or biologic therapy before the operation; (4) stage diagnosed with preoperative thoracic-abdominopelvic computed tomography (CT) with double contrast and intraoperative examination; (5) R0 resection (with no tumor cells at the margin) with D2 or more extensive lymph node dissection; (6) age from over 20 to under 80 years old. Exclusion criteria: (1) having received chemotherapy, radiotherapy, or immunotherapy before surgery; (2) distant metastases confirmed by postoperative pathological examination; (3) positive peritoneal cytology; (4) having definite diseases or abnormal laboratory test results; (5) having remnant gastric cancer; (6) having any condition not suitable for NIIC.

### Follow-up

Patients were followed up after the operation following the National Comprehensive Cancer Network (NCCN) guideline by clinical examination, carcinoembryonic antigen level, lung CT scan, and ultrasound of the abdomen every 3 months within 2 years after surgery. Follow-up was reviewed every six months for 2 to 5 years after surgery. Gastroscope was done 1 year after surgery and then repeated every 1 year in 3 years.

### Experimental design

A total of 1253 patients ultimately met the study criteria. In patients receiving NIIC, a fixed dose of 1000mg implant was placed adjacent to the tumor bed after surgical resection. Both the two groups were then further divided into subgroups including age, sex, BMI, lifestyle habits, chronic disease, pathological type, TNM stage, and differentiation grade.

### Statistics

IBM SPSS Statistics (version 26.0, IBM Inc., Armonk, NY, United States), and GraphPad Prism software (version 9, GraphPad Software Inc., San Diego, CA, United States) were used for statistical analysis. The baseline characteristics of patients were described using summary statistics. The continuous variables were presented as central tendencies (means or medians) and dispersions (standard deviations or interquartile ranges). For the group comparisons of the numeric variables, the Student’s t-test was used when the data were normally distributed, and the Mann-Whitney test for the variables in which distribution was not normal. Count indicators were described by numbers and percentages. The intragroup comparison was performed by using the chi-square test. Survival was estimated using the Kaplan–Meier method and differences between groups were estimated using the log-rank test. Cox regression ratio was used to obtain the crude hazard ratio and adjusted risk ratio for OS. *P* < 0.05 indicated a significant difference.

## Results

### Baseline information of the participants

In total, 314 patients with incomplete data and 115 patients with distant metastasis were excluded. 1253 patients were finally recruited and divided into two groups according to whether they received NIIC. There were 861 patients receiving NIIC and 392 patients not receiving NIIC. The flowchart of the study is shown in **Supplementary 1**.

The average age of the patients in the RC (receive chemotherapy) group was 62.1 ± 10.4 years, and that of the NRC (not receive chemotherapy) group was 62.2 ± 10.0 years. Furthermore, 79.3% patients were male in the NRC group while 74.1% of patients were male in the RC group. In the NRC group, patients with BMI below 18.5 were 17.6%, BMI between 18.5-23.9 was 70.9%, and BMI ≥ 24 was 11.5%. In the RC group, patients with BMI below 18.5 was 17.8%, BMI between 18.5-23.9 was 65.9%, and BMI ≥ 24 was 16.3%. In the NRC group, smoking and drinking patients accounted for 60.5% and 52.8%, respectively. In the RC group, smoking and drinking patients accounted for 58.7% and 53.2%, respectively. In the NRC group, adenocarcinoma accounted for 61.2%, squamous carcinoma accounted for 35.2%, and signet-ring cell carcinoma accounted for 3.6%. The baseline characteristics of patients were described using summary statistics. The detailed proportion of each tumor stage and differentiation degree in the two groups were also calculated. Results showed that the background data for all patients were relatively similar, suggesting that the two groups were comparable (**Table 1**).

**Table 1:** Baseline clinical characteristics of the patients.

Characteristic	No chemotherapy (n=392)	Received chemotherapy (n=861)	P value
**Age, n (%)**			0.31
Young (20-44)	79 (20.2)	203 (23.6)	
Middle (45-59)	165 (42.1)	379 (44.2)	
Old (≥60)	148 (37.7)	279 (32.2)	
**Sex, n (%)**			0.82
Males	311 (79.3)	638 (74.1)	
Females	81 (20.7)	223 (25.9)	
**BMI, n (%)**			0.47
<18.5	69 (17.6)	153 (17.8)	
18.5-23.9	278 (70.9)	567 (65.9)	
≥24	45 (11.5)	141 (16.3)	
**Smoking, n (%)**			0.52
No	155 (39.5)	356 (41.3)	
Yes	237 (60.5)	505 (58.7)	
**Drinking, n (%)**			0.59
No	185 (47.2)	403 (46.8)	
Yes	207 (52.8)	458 (53.2)	
**Chronic disease, n (%)**			0.43
No	135 (34.4)	323 (37.5)	
Yes (hypertension or diabetes)	257 (65.6)	538 (62.5)	
**Pathological Type, n (%)**			0.26
Adenocarcinoma	240 (61.2)	679 (78.9)	
Squamous Carcinoma	138 (35.2)	161 (18.7)	
Signet-ring cell Carcinoma	14 (3.6)	21 (2.4)	
**TNM stages, n (%)**			0.89
I	43 (11.0)	61 (7.1)	
II	148 (37.8)	312 (36.2)	
III	201 (51.2)	488 (56.7)	
**Differentiation Grade, n (%)**			0.73
Low	196 (50)	308 (35.8)	
Intermediate	103 (26.3)	294 (34.1)	
High	93 (23.7)	259 (30.1)	

The patients’ characteristics in group 1 and 2 are listed. No significant difference was found between the two groups (P > 0.05). The background data for all patients were relatively similar.

### Survival analysis

**Figure 1:**
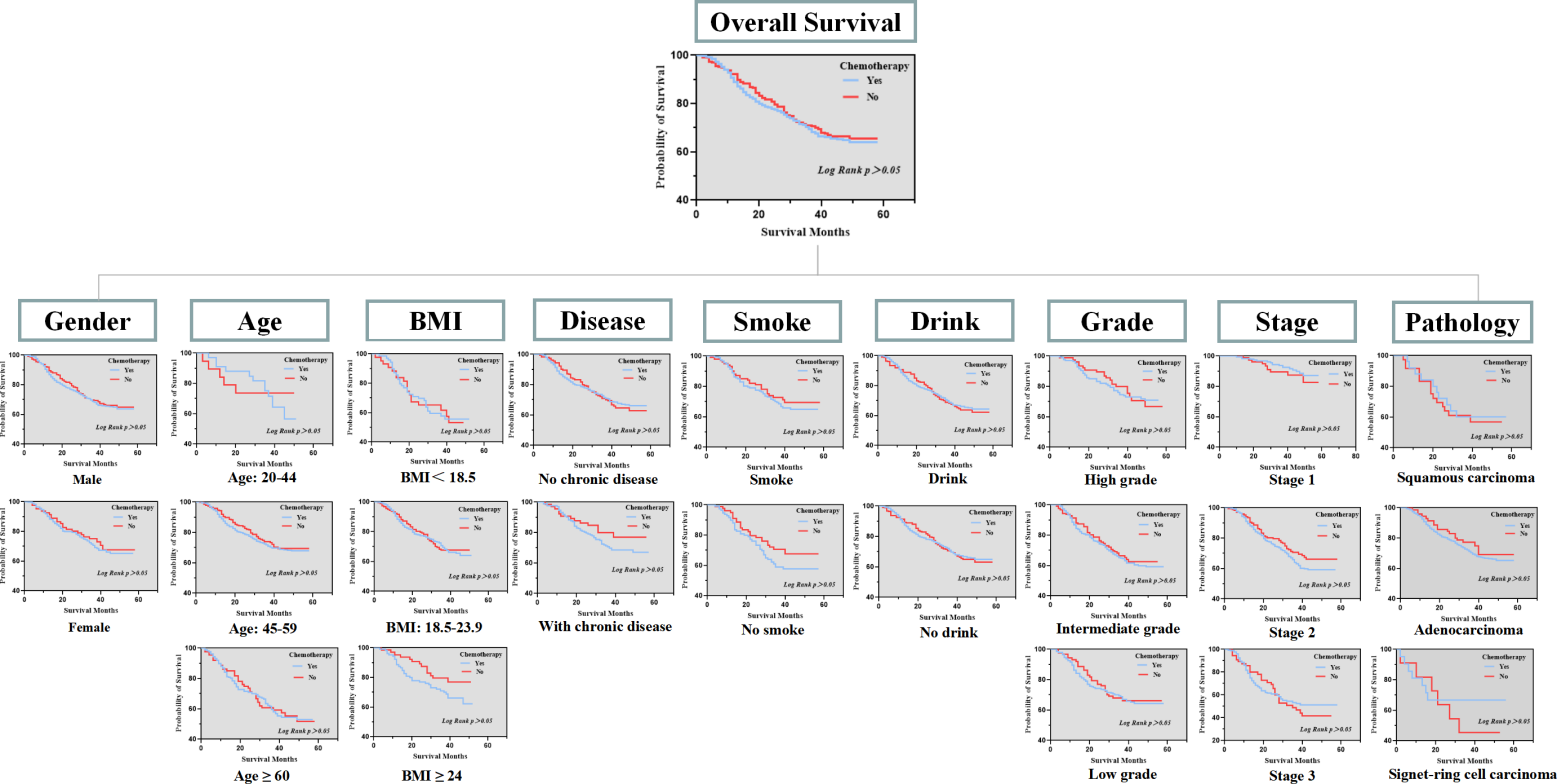
Overall survival between patients received NIIC treatment and patients without NIIC treatment in subgroups.

Subsequently, we conducted a K-M survival analysis to compare the overall survival between the two groups. No significant difference was found in the overall survival between patients who received NIIC treatment and patients without NIIC treatment (**Figure 1**). To further demonstrate the research conclusion, we repeatedly performed K-M survival analysis in various subgroups. The gender was categorized as male and female. Age was divided into three subgroups: 20-44 years old, 45-59 years old, and ≥ 60 years old. BMI was also categorized into three subgroups: <18.5, 18.5-23.9, and ≥ 24. Meanwhile, chronic disease, smoking, drinking, pathological type, differentiation grade, and TNM stage are separately divided into subgroups. Consistently, no significant difference was found between patients who received NIIC treatment and patients without NIIC treatment in each subgroup.

### Prognostic factors in gastric cancer patients

Ultimately, to further figure out the clinical variables associated with gastric cancer, the Cox proportional hazards model was applied to the analysis (**Table 2**). The multivariable Cox analysis also indirectly corroborated our conclusion, showing that the application of NIIC was not an independent risk factor affecting the prognosis. The multivariable Cox analysis also revealed that age, BMI, pathological type, TNM stage, and differentiation grade were independent risk factors affecting the survival of gastric cancer patients.

**Table 2:** Multivariate analysis of prognostic factors of OS in gastric cancer.

Characteristic	HR	95%CI	P value
Age			
Young (20-44)	1		
Middle (45-59)	1.035	1.048-1.011	<0.05
Old (≥ 60)	2.152	2.026-2.198	<0.05
Sex			
Males	1		
Females	0.956	0.642-1.422	0.823
BMI			
<18.5	1		
18.5-23.9	1.590	1.056-2.393	<0.05
≥ 24	1.970	1.679-2.458	<0.05
Smoking			
No	1		
Yes	1.272	0.669-2.416	0.463
Drinking			
No	1		
Yes	0.555	0.286-1.075	0.081
Chronic disease			
No	1		
Yes	1.185	0.576-2.439	0.645
Pathological Type			
Squamous carcinoma	1		
Adenocarcinoma	71.346	7.923-642.466	<0.05
Signet-ring cell	16.699	2.016-138.308	<0.05
TNM stages			
I	1		
II	7.874	3.245-8.019	<0.05
III	8.232	4.098-8.638	<0.05
Differentiation Grade			
Low	1		
Intermediate	0.838	0.567-1.240	<0.05
High	0.645	0.415-1.003	<0.05

## Discussion

Nowadays, gastric cancer remains one of the most deadly cancers in China (10, 11). Recurrence after gastrectomy, especially peritoneal recurrence, is a primary factor affecting the survival of patients with gastric cancer. Patients with gastric cancer and peritoneal carcinomatosis (PC) have a poor prognosis, with a median survival of 3.1 months without treatment (12). Studies demonstrated that extended resection including gastrectomy and peritonectomy combined with intraperitoneal chemotherapy (IPC) improves survival in patients (13–15). As one of the IPC treatments, NIIC has the theoretical advantage that injecting high concentrations of drugs directly into the abdominal cavity reduces systemic toxicity (16–18). Besides, the drug concentrations in the portal vein are also higher, which may be vital because the liver is a common site of metastasis (19).

Briefly, researchers have different opinions on the efficacy and safety of NIIC. In our real-world study, it was revealed that intraoperative intraperitoneal injection of normothermic chemotherapy drugs did not significantly ameliorate the outcomes of patients, which goes contrary to our previous perception. Notably, this conclusion is also tenable in our various subgroups (sex, age, BMI, chronic disease, lifestyle, tumor stage, differentiation grade, and pathological type). This real-world research conclusion indeed differs from partially published articles. The conflicting conclusions directly lead to the following questions: Despite NIIC being theoretically effective for patients with gastric cancer, is there an evident advantage in clinical practice? Is NIIC truly effective as we think? Should we abandon NIIC in gastric cancer if the benefits outweigh the disadvantages? These interlocking questions are worth deeply pondering due to the extensive application of NIIC in clinical.

Given that NIIC is defined by complex parameters, which include drug, dosage, concentration, inflow temperature, method, perfusion duration, and so on (20). Pharmacokinetic sufficiency and drug sensitivity are tightly related to NIIC efficacy. In different clinical models, the above parameters of NIIC may be different. Lacking the guideline to normalize NIIC may be responsible for the inconsistent results. For example, no guidelines recommend lobaplatin for NIIC in gastric cancer. Besides, different studies may focus on different patient populations. Some of these studies have mainly focused on patients with advanced gastric cancer, others may focused on patients in the early stage. Different characteristics of the study subjects may also result in different findings. Our study mainly included patients in TNM stage I-III. Moreover, some limitations of this study must be discussed. The data is not representative because this study is just a single-center sample study. The clinical outcome included the OS only, lacking the analysis of the relapses may also lead to compromised results. In addition, we did not collect clinical data related to postoperative complications, thus we could not ensure whether there were correlations between postoperative complications and NIIC. On the premise that NIIC could not improve the prognosis of gastric cancer patients, further explorations were not conducted to confirm whether NIIC would cause a series of side effects. The safety and serious adverse events rate of NIIC were not shown.

The chemotherapy regimen of NIIC for gastric cancer has not been established yet, but the various parameters including drugs, dosage, carrier solution and infusion methods are thought to be vital factors for the treatment efficacy. Treatment regimens combining multiple drugs are also expected to potentially yield superior efficacy. Meanwhile, the applied drugs are expected to meet certain demands. The selected drug (s) should be shown to be active *in vitro* and *in vivo* in the specific malignancy. The satisfactory pharmacokinetic profile and tissue permeability are also pivotal to achieving ideal treatment effects. The drug should be localized in high concentrations in the peritoneal cavity, avoiding diffusion through the peritoneal barrier to cause systemic toxicity. Highly volatile drugs are also unconsidered owing to the direct harm to the operating personnel. Besides, the carrier solution and infusion method are of great significance to the efficacy. Above all, more randomized controlled experiments and research are warranted to determine the appropriate indicators and the exact toxicity profile to realize favorable treatment outcomes.

## Data availability statement

The raw data supporting the conclusions of this article will be made available by the authors, without undue reservation.

## Ethics statement

Ethical review and approval was not required for the study on human participants in accordance with the local legislation and institutional requirements. Written informed consent from the patients/participants was not required to participate in this study in accordance with the national legislation and the institutional requirements.

## Author contributions

MJ, WC, and JC were responsible for the acquisition and analysis of data, had full access to all of the data in the study, and take responsibility for the integrity of the data and the accuracy of the data analysis. BC and MX have revised the original draft. GC conceived the idea and revised the manuscript. All authors were responsible for the interpretation of data, the drafting, and critical revision of the manuscript for important intellectual content. All authors contributed to the article and approved the submitted version.
