# Supplementary figures and images for: A real-word study: is normothermic intraoperative intraperitoneal chemotherapy impactful as we expect?
Source: Front Oncol. 2023 Jul 4;13:1172782. doi: 10.3389/fonc.2023.1172782 (PMC10352766; doi:10.3389/fonc.2023.1172782)

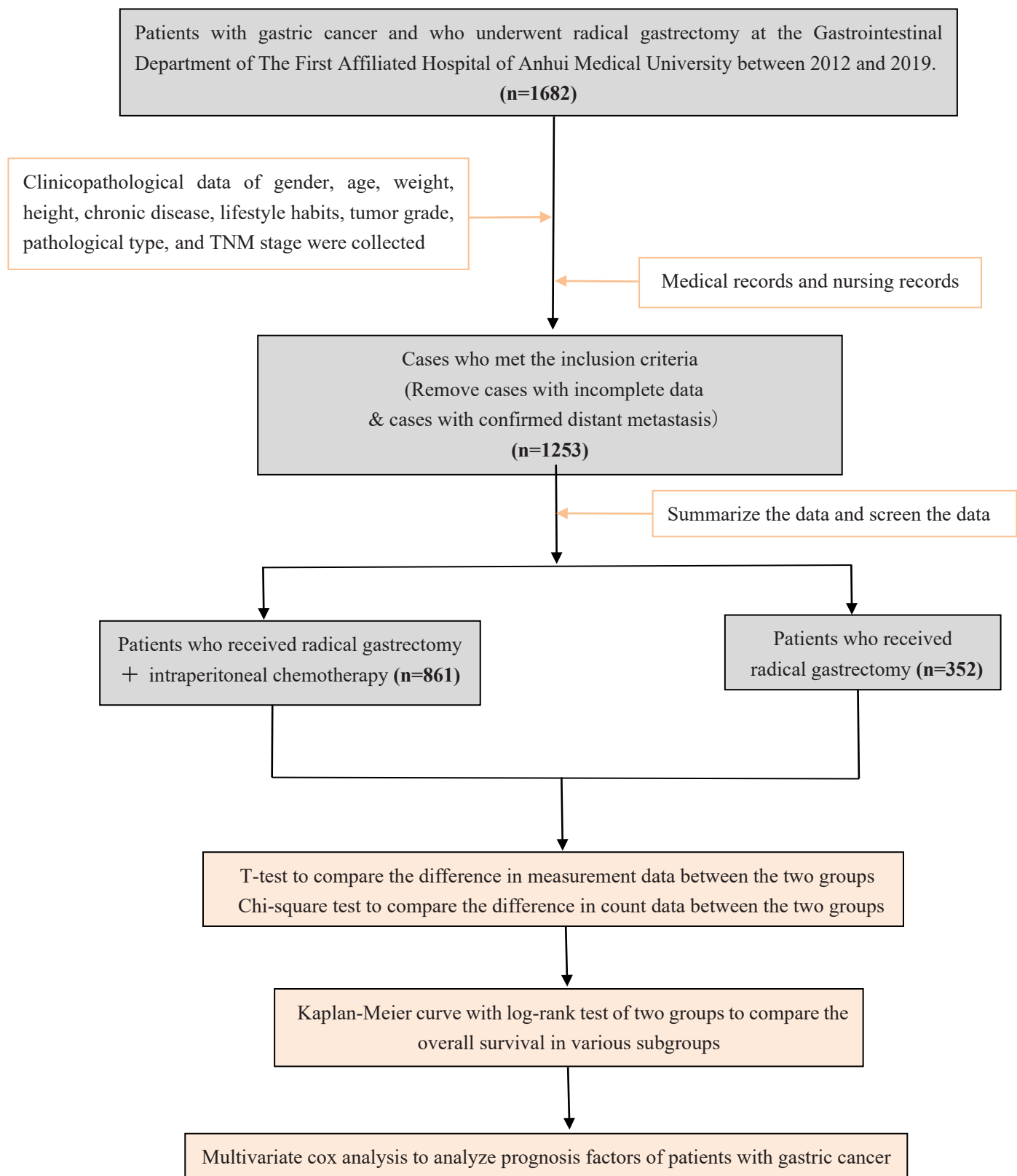

**Supplementary 1. Flowchart of the original research**

Supplement: Supplementary file 1 [file Image_1.pdf]
